# Content Analysis of Student Essays after Attending a Problem-Based Learning Course: Facilitating the Development of Critical Thinking and Communication Skills in Japanese Nursing Students

**DOI:** 10.3390/healthcare5030047

**Published:** 2017-08-22

**Authors:** Tomoya Itatani, Kyoko Nagata, Kiyoko Yanagihara, Noriko Tabuchi

**Affiliations:** Division of Nursing, Faculty of Health Science, Institute of Medical, Pharmaceutical and Health Sciences, Kanazawa University, 5-11-80 Kodatsuno, Kanazawa 920-0942, Japan; nagata@mhs.mp.kanazawa-u.ac.jp (K.N.); kyana@mhs.mp.kanazawa-u.ac.jp (K.Y.); tabuchi@staff.kanazawa-u.ac.jp (N.T.)

**Keywords:** first-year education, problem-based learning, critical thinking, communication, nursing education

## Abstract

The importance of active learning has continued to increase in Japan. The authors conducted classes for first-year students who entered the nursing program using the problem-based learning method which is a kind of active learning. Students discussed social topics in classes. The purposes of this study were to analyze the post-class essay, describe logical and critical thinking after attended a Problem-Based Learning (PBL) course. The authors used Mayring’s methodology for qualitative content analysis and text mining. In the description about the skills required to resolve social issues, seven categories were extracted: (recognition of diverse social issues), (attitudes about resolving social issues), (discerning the root cause), (multi-lateral information processing skills), (making a path to resolve issues), (processivity in dealing with issues), and (reflecting). In the description about communication, five categories were extracted: (simple statement), (robust theories), (respecting the opponent), (communication skills), and (attractive presentations). As the result of text mining, the words extracted more than 100 times included “issue,” “society,” “resolve,” “myself,” “ability,” “opinion,” and “information.” Education using PBL could be an effective means of improving skills that students described, and communication in general. Some students felt difficulty of communication resulting from characteristics of Japanese.

## 1. Background

Active learning is defined as an educational activity that requires students to have first-hand experience and to think for themselves about their actions [1]. Active learning was first introduced in Japan in the 1990s, and its importance in university education has continued to increase [2,3] because Japan had seen a diversification of values stemming from societal changes. For example, family composition has diversified as single households and nuclear families increase. Additionally, employment patterns have become more diverse, resulting from an increase in non-regular employment. As a whole, society has also become increasingly informed owing to the ubiquity of the internet.

In our complex society, we all need the ability to collect information, accept variable values, judge subjectively, and resolve issues with other people. From this point of view, it is important for each person to develop the skills to examine issues from various perspectives [4]. According to “The Occupational Consciousness and Knowledge, Essential Abilities and Skills for College Students’ Survey” conducted by the Japanese Federation of Economic Organization, “subjectivity,” “communication,” “ability to execute,” and “teamwork and cooperativeness,” were ranked as the top skills that companies look for in new workers [5]. Owing to the desirability and importance of such skills, the authors’ university has set the following educational goal: “By encouraging learning through direct experience, students acquire certain basic academic skills and comprehensive perspectives during their undergraduate courses.” In order to realize this goal, the “Kanazawa University Global Standard” (KUGS) was established. KUGS is an educational policy that concretely shows the human resources Kanazawa University will cultivate in undergraduate courses. To actively fulfill this mission in the international community, Kanazawa University aims to nurture human resources into becoming leaders of a knowledge-based society who have the ability to face associated difficulties. One of the five KUGS items is, “express ideas and values.” The aim of this is for students to acquire “the ability to clarify their thoughts and ideas using words and charts, and express their own sensibilities and emotions, behaviors, and perspectives underlying their thoughts or expressions” [6]. We conducted a class on debate theory and presentation using the problem-based learning (sometimes known as project-based learning: PBL) method. This kind of active learning is part of a general introductory undergraduate course for first-year students majoring in education. PBL is said to be an effective way of linking practice and theory within the university learning environment [7]. A previous study of medical students suggested that education using PBL was effective in improving self-ratings on general competencies and interpersonal skills [8]. In nursing education, PBL began to be used as a decision-making beginning at the end of the 1980s [9].

Active learning, originally developed by McMaster University in Canada, was introduced into Japanese nursing education in the late 1990s [10]. The purpose of introducing active learning was to improve self-development in education and to support nurses with self-reflection and problem-solving abilities in response to societal changes. PBL is said to be effective in the acquisition of critical thinking [11]. Critical thinking is defined as “intentional self-control judgments including interpretation, analysis, evaluation, reasoning, and contextual consideration” [12]. In the 2000s, the number of established four-year nursing baccalaureate programs was increased in Japan and the curricula design increasingly focused on critical thinking [13]. Hashimoto et al. examined the relationship between PBL and communication for university students and revealed that it has an effect in communication skills training [14]. In nursing education, the practice of having students work in small groups to foster critical thinking and communication skills has been actively adopted. However, in traditional nursing education methods, it is difficult to say that learner-led, self-development education has been provided. Subsequently, even after the introduction of tutorial education, PBL has not been established in these programs.

Numerous studies evaluating students’ critical thinking abilities have been conducted in various countries around the world. Carter et al. reviewed previous studies using the Critical Appraisal Skills Program Tool, and found inconsistent reliability and validity [15]. Critical thinking has also been studied using qualitative methods: Bittencourt et al. conducted an exploratory descriptive study on the application of a clinical case to identify critical thinking skills with seven nursing students and capture their justifications for decisions in the nursing diagnosis process, and performed content analysis to evaluate these data [16].

Against this background, this study aimed to developed students’ critical thinking and communication skills by providing classes incorporating PBL, and incorporating a post-class student essay task about critical thinking and communication. The purposes of this study were to analyze the post-class essay, describe the status of students’ logical and critical thinking after attended a PBL course.

## 2. Methods

### 2.1. Setting

This study was conducted in several steps. First, the author designed and conducted the course of classes using the PBL method named “Kanazawa University debate skills and presentation”. The classes took place from June 15 to August 3, 2016. Next, the authors received post-course essays as an assignment from the students. Then, the authors explained the study to the students and recruited participants. Finally, the authors analyzed those of the submitted essays that were written by participants who agree to participate in the research. This analysis was conducted between 1 October and 30 November 2016.

### 2.2. Sample and Recruitment

The classes were mandatory for students who entered the nursing program in 2016. Eighty-one students aged 18 or 19 were enrolled in the course, which consisted of eight 90-minute classes. Almost all students participated in every class and no students dropped out. After completing all the courses, every student submitted the post-essay following the course as an assignment. After that, the authors explained the study to the students and seventy-one students agreed to participate in the research.

### 2.3. Data Collection

The essays were created in Microsoft Word and submitted to a website installed on the on-campus network. The authors downloaded the essays and deleted the participants’ names.

### 2.4. Ethics

The authors distributed informed consent forms and information pertaining to the purpose and methodology of the study in advance of student participation. Participants were also verbally informed about the ethical considerations including deleting the participant’s name at the time of essay analysis for anonymity, voluntary participation, and participants’ right to withdraw without penalty. All participants provided written informed consent prior to participation. This study was approved by the University of Medical Ethics Review Committee of Kanazawa University (approval number 724-1).

### 2.5. Class Structure and Post-Course Essay Task Procedure

In this study, the course consisted of debate and PBL. The course grade was calculated based on degree of class participation, enthusiasm in discussions, preparation of a handout, creation of a PowerPoint presentation, and the post-course essay. The aim of the essay was to evaluate the formation of logical and critical thinking. The classes consisted of the following four stages. (1) During the first class, five or six students formed groups and each group chose one topic from eight pre-prepared societal topics that they were likely to have opinions on including “the risk of dating violence,” “the danger of religious solicitation,” “the voting behavior of 18-year-olds,” “the trap of social networking services,” “the dangers of binge drinking,” “opinions and arguments about whether the constitution should be reconciled,” and “are nuclear power plants necessary?” Each group addressed the same selected theme throughout the course. (2) Students discussed and shared their acquired information and knowledge in the small groups in the second to fourth classes (group work). (3) Students in each group integrated their knowledge with one another and created a PowerPoint slide that was presented to the other students in the fifth to seventh classes (group work). (4) During the final class, each group gave a PowerPoint presentation to the rest of the class and facilitated a question and answer session.

The authors played a supporting role as tutors during the classes by assisting with the development of students’ thinking processes and helping them to expand their knowledge; however, they did not provide information or present their own opinions. The aim of the group work was to enhance critical thinking abilities through exposure to different values, peer discussion, active listening, communicating opinions, and critical examination of not only themselves, but others.

After the course, the authors gave the students two tasks covering three themes. Task 1 asked students to prepare an essay on the topic, “What kind of skills and attitudes do you think are necessary to resolve any kind of social issue?” In Task 2, students responded to two questions: “What do you need to do in order to clearly convey your thoughts and opinions to others?” and “What is difficult when you tell others about an issue?”

### 2.6. Data Analysis

The authors analyzed the submitted essays using both Mayring’s methodology for qualitative content analysis [17] and text mining.

Qualitative content analysis is an approach involving empirical, methodical, controlled analysis of texts without quantification, intended to analyze all kinds of recorded communication [18]. The methodology was developed in detail by Mayring [17]. Mayring’s methodology was used to extract codes through explicative content analysis; then, content analysis was summarized to develop categories and subcategories. In this study, the authors extracted sentences describing students’ thoughts about PBL and communication while retaining the meaning, context, and original words where possible. Next, the authors confirmed the similarity of the meaning of the initial codes, assigned subcategory names, further integrated similar subcategories, and assigned category names. The credibility of the analysis was validated by three co-authors, who read the descriptions separately under supervision from qualitative research experts. The authors judged the credibility of the study based on previous research [19] according to how well the category covers the data, whether relevant data are inadvertently excluded, and whether irrelevant data are included.

Text mining analyzes large amounts of text data from various perspectives and with various goals [20]. In recent years, studies using text mining have been common in clinical fields [21], including nursing [22]. On the other hand, issues with the method have also been pointed out. Goodwin et al. conducted a study on building knowledge in nursing and concluded that use of data mining will make significant progress only if important data that incorporate expert nurses’ knowledge are made available in the clinical information systems text mining in health research draws on [23]. This suggests that it is difficult to build knowledge with text mining alone. In our study, the authors tried to extract students’ knowledge using text mining and qualitative content analysis at the same time. In text mining, the authors first imported the essay content as text data into a computer, classified the same clauses used in essays when broken into words, and classified the words into parts of words (morphological analysis). Second, after analyzing the trends and features of the classified words, frequently occurring words and keywords were extracted, and their frequency and simultaneous occurrence relationships were analyzed. Finally, analysis of the characteristics of the words, connections between words, and essay content were analyzed in concert with the results of qualitative data analysis. KH Coder (ver. 2.00) was used for analysis. Analysis was initially performed in the Japanese language, then results were translated into English when preparing this research paper. English language translations were verified by bilingual research collaborators.

## 3. Results

### 3.1. Qualitative Analysis

In the qualitative analysis of the essays written by 71 students, 15 categories, 32 subcategories, and 71 codes were extracted from the three themes: What kind of skills and attitudes do you think are necessary to resolve any kind of social issue? What do you need to do in order to clearly convey your thoughts and opinions to others? and What is difficult when you tell others about an issue? Categories, subcategories and codes are shown in Table 1, Table 2 and Table 3. The author described each category with respect to each theme included in the post-essays. Categories are indicated as ( ).

#### 3.1.1. What kind of skills and attitudes do you think are necessary to resolve any kind of social issue?

In this theme, seven categories were extracted as below.

(Recognition of diverse social issues) Regarding the various identified social issues, students described various problems such as politics, economics, and medical treatment. They also expressed the opinion that issues change as society changes, and pointed out that social issues are not uniform.

(Attitudes about resolving social issues) Students stated that there was a need to have a strong will and mission. In addition, they stated that it was necessary to develop solutions based on various people’s standpoints.

(Discerning the root cause) Students stated the importance of continuing to wonder “Why?”and having a detailed interest in topics. Furthermore, they realized that it is important to not only accurately investigate and grasp the information but also to understand information by acting upon it.

(Multi-lateral information processing skills) The ability to collect information multi-laterally includes not only collecting information but also the ability to capture problems without prejudice. Students indicated that it is important to identify the essence of the problem in order to obtain accurate information. Additionally, they thought that it was necessary to look at issues from different perspectives.

(Making a path to resolve issues) Here, students stated that goal setting needed to be clear and feasible.

(Processivity in dealing with issues) In this case, students highlighted the importance of the ability to “receive” and “send.” They also recognized how they could share information with others by raising a problem. In the “problem-solving cycle,” they noted the necessity of repeating the process of questioning, understanding and execution. Additionally, they stated that it was necessary to examine the problem, think, disseminate opinions, and establish a group.

(Reflecting on things) Students realized that taking steps for reflection was a way to review their thought process as well as have doubts and think about better solutions. They stated that they had the advantage of being able to organize their own thoughts by knowing themselves.

#### 3.1.2. What do you need to do in order to clearly convey your thoughts and opinions to others?

In this theme, five categories were extracted.

(Simple statement) To narrow down the statement, students were aware of the need to talk to an appropriate extent with their opponents. In order to clarify their conclusion, they stated that it is necessary to first express their thoughts and arrive at a concise conclusion.

(Robust theories) Students were aware of the need to infer the causal relationship between the problem and cause and to clarify the basis of the problem by accurately grasping the information.

(Respecting the opponent) Students realized that it was important to state their opinions without denying those of others, consider others’ feelings, and accept others’ opinion.

(Communication skills) Students stated that it is necessary to communicate in a way that encourages dialogue speak so as not to become unilateral and to confirm whether it is transmitted using approaches such as eye contact.

(Attractive presentations) Students stated that to attract others’ interest it is necessary to use words and images that are familiar to their opponents, create expressions tailored to the story, and to speak with confidence. In addition, they thought that it was important to create meaningful PowerPoint presentations and to utilize appropriate pauses to prevent others from getting bored.

#### 3.1.3. What is difficult when you tell others about an issue?

In this theme, three categories were extracted.

(Difficulty identifying others) Students stated that there was difficulty determining if opponents grasped comprehension based on their reactions. They felt there was the possibility of misunderstanding between speakers and listeners because each person understands the story in a different way. Additionally, because the opponent may be indifferent, they stated the importance and difficulty of keeping the listener’s interest.

(Difficulty of presentations) This category signified students’ difficulty talking while confirming the opponent's reaction with eye contact to ensure that the information was conveyed to others. They also stated the importance and difficulty of sorting and choosing the information they wanted to convey, and developing an ingenious way to communicate.

(Difficulty from lack of confidence and communication gaps) Students felt it was difficult to convey information because they were not confident in their thoughts and opinions. They also felt it was difficult to convey knowledge because there were differences in the generation, environment, and the amount of knowledge between themselves and the opponent.

### 3.2. Text Mining

**Figure 1:**
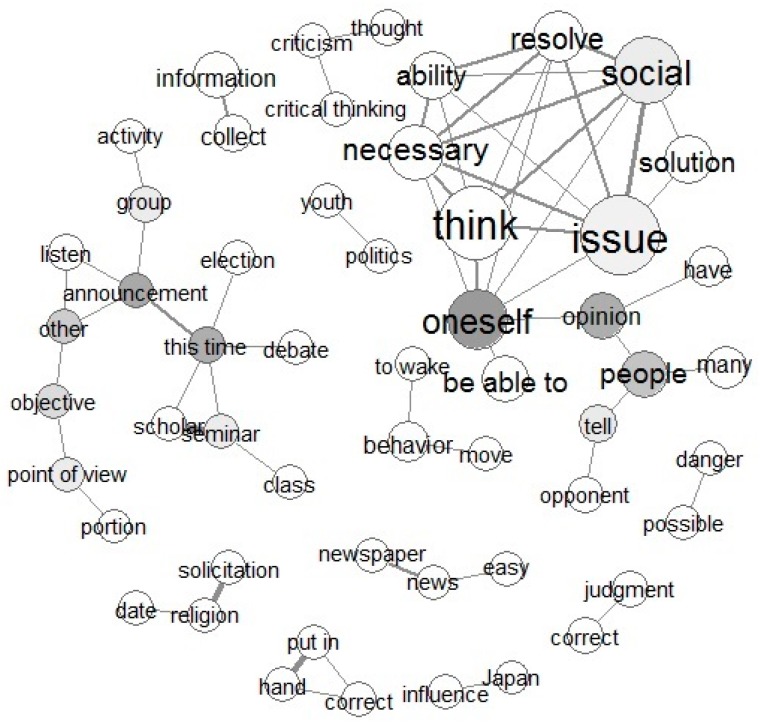
Co-occurrence words network of Task 1 responses to “What kind of skills and attitudes do you think are necessary to resolve any kind of social issue?”.

**Figure 2:**
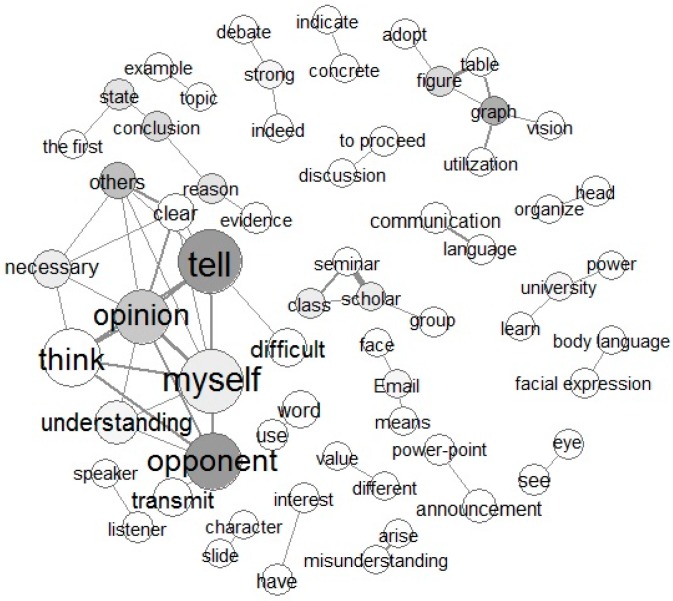
Co-occurrence words network of Task 2 responses to “What do you need to do in order to convey your thoughts and opinions to others?” and “What is difficult when you tell others about an issue?”.

Words extracted by text mining and their frequency of use are described blow. The results of the co-occurrence networks are shown in Figure 1 and Figure 2.

The total number of sentences from Task 1 (“What kind of skills and attitudes do you think are necessary to resolve any kind of social issue?”) was 866, and the total number of paragraphs was 124. The frequency of appearance of four words, “issue,” “society,” “necessary,” and “resolve” were ranked most highly. This result seems to have occurred because these words were used in the essay task theme and were cited in the essay text. Other extracted words that appeared more than 100 times were, “myself,” “solution,” “ability,” “able,” “people,” “opinion,” and “information.” The results of text mining of frequently extracted words suggests that students recognized the necessity of information literacy. These results support those of our qualitative data analysis.

The “co-occurrence network” figure expresses the frequency of words and co-occurrence of two or more words. Figure 1 shows the co-occurrence network for Task 1. In the co-occurrence network diagram, strong co-occurrence relationships are indicated by thick solid lines, and words with a high number of occurrences are displayed in large circles. Words closer to the center of the structure are indicated in darker gray. The network revealed words including “think,” “social,” “issue,” “resolve,” and “necessary,” which occur frequently with “oneself” at the center of the network. Based on this context, the network suggests meanings such as “it is necessary to think oneself in order to resolve social issues.” Additionally, a network including “tell” and “opponent” with “people” and “opinion” at the center was formed, and was connected to a network centered on “oneself.” The group centered on “announcement” consisted of two groups, one including words such as “other,” “objective,” and “point of view.” The other group included words such as “this time,” “seminar,” and “debate.” Also, “information” was linked to “collect,” leading to “collecting information.” Other networks included context on PBL themes. For example, a network of “solicitation”­“religion”­“date” was derived from “the danger of religious solicitation,” a PBL theme.

Task 2 included two themes: “What do you need to do in order to clearly convey your thoughts and opinions to others?” and “What is difficult when you discuss an issue with others?” The total number of sentences for this task was 858, and the total number of paragraphs was 134. The most frequently used words (“myself,” “tell,” “think,” “opponent,” and “opinion”) were partially explained by the fact that they were in the essay title and that participants restated this title within the essay. Other extracted words that appeared more than 100 times were, “understanding,” “important,” “transmit,” and “difficult.” Extracted words with frequencies of 50 or more were “necessary,” “word,” “clear,” “speak,” “others,” “people,” and “feel.” It is thought that the frequently occurring words “tell,” “opponent,” “others,” and “opinion” occurred because the participants quoted the theme title. Linking of these words was also shown in co-occurrence networks. For instance, a network of “the first”­”state”­”conclusion”­”reason”­“network” was formed. From these words, the context of “to state conclusion first” appeared to occur. This content was also found in the qualitative analysis as a way to clearly convey an opinion. A network centered on “graph” and “figure” linked to “table,” “adopt,” and “utilization” was also established. Since words forming this network are low in occurrence frequency, they are expressed in small circles. It is thought that this network refers to the tables and figures used when communicating opinions. A network of “myself”­”opinion”­”tell”­”opponent”­“think” was formed. From these words, the meaning “tell opinion to opponent” appeared to occur. Also, this network is linked to “understanding” and “clear,” yielding the meanings “tell opinion clearly” and “understanding opponent” appeared to occur. The term “body language,” which was used in the essays, is closely related to “facial expression,” because the two terms are linked in the co-occurrence network.

## 4. Discussion

### 4.1. Thinking about Social Issues and Ethics

Critical thinking is said to be associated not only with knowledge skills, but also emotional aspects [12,24]. In this study, qualitative analysis suggested that students were aware of the diversity of social issues and the importance of different attitudes. Currently, there are many diverse social issues present in Japan that may be commonly recognized by Japanese individuals. Naturally, students would have recognized the various social problems in Japan before taking classes involving PBL. Thus, it would be reasonable to regard the “various social issues” described by the students as descriptions of their existing knowledge of the issues, rather than something that they learned in the classes. Additionally, as pointed out by students, social issues often involve ethical issues. For example, the issue of nuclear power plants, one of the themes of the course, includes the ethical problem about how the stable supply of energy often conflicts with residents’ security. The problem of nuclear power plants is an issue that has been widely discussed in the country since the occurrence of a high-profile nuclear accident in 2011 [25]. Even in the context of social issues, medical care often includes ethical concerns. For example, Dr. Yamanaka of Kyoto University received the Nobel Prize for research on iPS cells in 2012 [26]. Subsequently, people understood that there were favorable discoveries that contributed to the advancement of medicine, while at the same time discussing issues related to bioethics. Although participants in this study were first-year students, they might be sensitive to ethical issues because they aimed to become nurses. Although they will touch on ethical issues while studying nursing science, attitudes and consciousness of ethical problems among first-scholars through PBL are desirable. In this respect, discussing social issues using PBL should have been beneficial. Discussion during PBL often caused a conflict of opinion among students. As a result, the students discovered the ethical nature of social issues, while at the same time discovering the attitudes necessary to deal with these issues such as clarifying their opinions and considering the positions of others. According to the Japan Nursing Association, morality is often used in small groups such as individuals and families, whereas ethics is often used extensively in individuals’ relationships to society; thus, the direction of treatment in the medical field is determined by ethical judgment criteria rather than morality [27]. In other words, it is essential to nurture ethics by making young scholars think about ethics, and in this sense, should be a beneficial effect of PBL.

### 4.2. Information Literacy

In Japan, the number of Internet users is over 100 million and the population penetration rate is 91.0% [28]. The recent increase in the penetration rate of smartphones has also increased, reaching 84% [29]. Consequently, information can easily be accessed in Japanese society. Even in this course, most students used the Internet via personal computers or smartphones to collect information. During qualitative analysis, students recognized that it is necessary not to blindly accept this information when dealing with social problems, and to determine the fundamentals by pursuing their interests. This awareness is important, as it can help them avoid confusion caused by erroneous information in an information-rich society. As the enormous amount of information overflows, it is necessary to ascertain what information is valid and accurately grasp the truth in order to accurately capture information. As previously mentioned, when dealing with ethical issues, we also need to consider both our own thoughts and others’ opinions. Therefore, more than one aspect of the information needs to be categorized; instead, categorization should occur in a multifaceted manner, a process that was recognized by students. As mentioned above, students had learned that information literacy is necessary for resolving social issues, which is considered evidence of the effectiveness of PBL.

### 4.3. Critical Thinking

One of the concepts of critical thinking is “processivity.” Students recognized the necessity of processivity, which includes setting goals and priorities in advance, receiving and sending information, and debating when trying to resolve social issues. They also recognized the necessity of looking back on the process after problems were solved. This series represents “processivity.” Critical thinking involves thinking that includes clarification of information, examination of speculative foundations, reasoning, behavioral decisions, and problem-solving [11]. The corresponding students’ recognitions are (multi-lateral information processing skills), (discerning the root cause), and (making a path to resolve issues). In addition, students recognized that looking back was necessary. Specifically, they described how they could know and categorize their own thoughts through reflection on previous ideas. This represents “reflection,” which is the main concept in critical thinking [30]. Reflection is especially characterized by the thoughts of professional practitioners such as nurses [31].

As previously noted, the series of concepts students recognized is more than problem-solving ability; instead, it is indicative of critical thinking. Therefore, it is suggested that one of the lessons students learned through PBL might have had a certain effect on nurturing their critical thinking.

### 4.4. Communication with Individuals

The answers to the question “What do you need to do in order to clearly convey your thoughts and opinions to others?” suggested that students broadly recognized the importance of theory and communication skills. Specifically, students recognized the importance of robust theory that showed causal relationship or evidence as well as the simple manifestations that are required in theory composition. These ideas are part of scientific thinking, including critical thinking. While students exchanged opinions with each other while working on PBL, it appears that that logical thought was used to accurately convey their opinions at that time. Students also thought of social problems as being “diverse.” When discussing such diverse issues, conflicting opinions were often encountered. Under these circumstances, it is necessary to advance constructive discussions by demonstrating respect for the partner. Here, we obtained two suggestions for the effects of PBL implementation. One is that PBL could have a positive effect on respecting the partner, which is also crucial in nursing. The other is communication, and it is suggested that PBL is also effective in improving communication skills. In other words, students tried and erred in communicating with other students, and learned how to communicate clearly through PBL.

### 4.5. Presentations

As shown in “attractive presentations” in Table 2, students learned how to make a successful presentation by practicing this skill. Although students mentioned the presentation methodology, the content was universally important in interpersonal communication. This suggests that presentations are effective as a kind of active learning to improve students' communication skills.

### 4.6. Effectiveness of Education Using PBL

Students experienced classes and presentations using PBL and debate. In summary, it was suggested that, through this education, students learned how to handle information, attitudes, and communication skills to facilitate the exchange of opinions with others and that education using PBL is an effective means of improving those skills. We have described the positive effects of PBL. On the other hand, the answer to the question of “What is difficult when you tell others about an issue?” leads us to suggest improvements for future education.

### 4.7. Communication Difficulties

Students mentioned the necessity of confirming the comprehension level of others’ as a communication skill. However, they perceived this as difficult to accomplish. They said that they cannot perceive others’ comprehension level while concentrating, or when the others’ responses are restrained. In addition, students thought that it was necessary to adapt to their partner during communication, but recognized that sharing interests and emotions was difficult. Thus, it is suggested that important matters in communication can be difficult in practice. The cause of this is differences in generation, age, environment, and extent of knowledge compared to the opponent. However, providing nursing care to people of different ages, environments, and knowledge occurs on a daily basis. In this situation, it is necessary to understand others’ comprehension level, especially that of patients, and to share their interests and feelings. Therefore, the challenge is determining what kind of education should be implemented so that smooth communication can be established between different parties.

With respect to presentation skills, students recognized the importance of adjusting facial expressions to the audience and speaking with an organized plan. However, although it is necessary, they recognized that it was difficult to carry out, reflected in codes such as “it is difficult to confirm with eye contact” and “it is difficult to organize stories”. In particular, eye contact might be very important for the Japanese. Consistently, Ohbuchi (1994) essayed a strong tendency for confrontation avoidance as a Japanese communication style [32]. The results of this study also showed that confusion occurred and others’ reactions were not understood because students avoided confrontation and paid attention to others to avoid hurting others. This is also expressed in codes as “difficulty from lack of confidence and communication gaps”; specifically, it is recognized as “understanding varies depending on the person”, or “lack of confidence in one’s thoughts and opinions”. Furthermore, it can be inferred that this is linked to “fear of speaking and hesitation”. The characteristics of Japanese culture include cooperativeness and compassion for others, which may well be regarded positively. However, smooth communication is necessary in dealing with complicated social issues or providing nursing care. Therefore, it is necessary to consider educational methods to improve communication skills based on the communication characteristics of Japanese people.

In the educational steps for critical thinking, there are roughly four approaches to educational methods [33]. The first “general approach” is universal in the field. Specifically, it is a method of teaching doctrines in classes such as logic and ethics. The second “infusion approach” is to explicitly teach critical thinking skills in specialized education such as nursing. The third is the “immersion approach,” in which the learner is deeply involved in a specialized subject. The fourth is the “mixed approach,” which combines the general approach with either the infusion or immersion approaches. Kusunoki, a pedagographer, stated that critical thinking was logical thinking based on a goal with conscious introspection (reflection) [34]. She also stated that critical thinking led to evidence-based nursing and acted as a foundation for nurses’ learning and research. Although the PBL themes used in this study were not limited to nursing, students were aware of the fundamental necessities of critical thinking such as information collection and logical thinking. In other words, students demonstrated universal thinking by using a wide range of themes not limited to nursing. Therefore, PBL is effective for achieving a general approach, which is the first stage of critical thinking. It is also expected that this will lead to critical thinking in the specialized field (the infusion approach as well as the immersion and mixed approaches). The results of the current study suggest that practicing education using PBL has the effect of nurturing students’ communication skills. However, students found it difficult to communicate with others. We found that there were characteristics of Japanese people, such as “respecting others” and “fitting behavior to the others.” As clinical nursing involves interpersonal aid for patients, interpersonal communication skills must be high. In this sense, it is beneficial to implement PBL to scholars at the onset of their education. However, understanding the characteristics of the Japanese students, it is necessary to conduct education so that it will not become a barrier to communication. Specifically, we should educate students by attempting to prevent misunderstandings with others and facilitating confidence in their opinions. These are points to be clarified in future research. Especially as globalization progresses, the combination of communication skills and self-confidence is becoming increasingly necessary.

### 4.8. Limitation and Future Study

The limitation of this study is that there was no group of students who wrote essays on the required themes without PBL education. Consequently, the study should be conducted using a control group to further examine the effectiveness of PBL. Also, due to the structure of the university curriculum, class time was limited to 90 min, and due to the limitation of the number of faculty members, the groups were somewhat large at five or six members. However, if students had spent more time on PBL, or tried it in smaller groups, the results may have been different. Even in this respect, however, it is necessary to compare groups with different constituent members, and classes with different contents.

## 5. Conclusions

After PBL, using various social issues as themes, students discussed the importance of developing ethics, information literacy, critical thinking, and communication skills. Education using PBL could be an effective means of doing all these things. Students described about their communication skills after debating with other students. Some students felt the difficulty of communication, and the difficulty is thought to be attributable to the characteristics of the Japanese as “matching with the other party.” This conclusion was derived from the results of not only the concept analysis, but also text mining.

## Figures and Tables

**Table 1:** Content categorization of Task 1 responses to “What kind of skills and attitudes do you think are necessary to resolve any kind of social issue?”.

Category	Subcategory	Code
Recognition of diverse social issues	Social issues are varied	There are various problems such as politics, finance, medical treatment
		Challenges change as society changes
	Social issues involve ethics	There are also issues involving ethics
		Problems arise owing to the development of science and technology
Attitudes about resolving social issues	Having a strong will	Strong will and mission
		Determining one’s will
	Thinking about the opponent	Working on solutions by assuming the role of various people
		Comparing one's position and the opponent’s position
Discerning the root cause	Pursuing issues with detailed interest	Always wondering “why?”
		Dedicated interest in topics
		Facing and pursuing issues
	Not trusting all available information	Examination and accurate understanding
		Importance of confirming finding appropriate information
		Summarizing the fundamental problem
Multi-lateral information processing skills	The ability to diversely collect information	Multi-lateral, multi-angle information collection
		Capture issues without prejudice
	The ability to categorize information	Discernment in obtaining accurate information
		Restricting social issues to one’s own field
Making a path to resolve issues	Setting appropriate goals	Clear goal setting
		Realistic goal setting
	Establishing priorities	Prioritizing and assigning issues
		Executing in order
Processivity in dealing with issues	Sending and receiving information	The ability to “receive” and “send”
		Sharing by sending issues
	Problem-solving cycle	Repeating question - understanding - execution
		See, think, disseminate opinion, establish a group
Reflecting	Taking steps of reflection	Reviewing and reconsidering one’s process
		Having doubts and considering better solutions
	Knowing oneself	Knowing one’s own thoughts
		Benefits of organizing one's thoughts

**Table 2:** Content categorization of Task 2 responses to “What do you need to do in order to clearly convey your thoughts and opinions to others?”.

Category	Subcategory	Code
Simple statement	Narrowing down the statement	Narrowing down what I want to say
		Speaking to the appropriate extent depending on the opponent
	Conclusion first	Expressing one's thoughts first
		Concluding in a simple manner
Robust theory	Showing causal relationships	Considering the root cause of the issue
		Showing the causal relationship between issues and causes
	Showing evidence	Examining and accurately grasping the information
		Concrete communication with clear evidence
Respecting the opponent	Non-referee attitude	Expressing opinions without denying others
		Not imposing one's opinion
	Understanding the opponent	Thinking about the opponent's feelings
		Accepting the opponent’s opinion
Communication skills	Ingenuity in clearly conveying opinions	Creating a dialogue
		Speaking clearly as to not cause misunderstandings
	Ingenuity in checking the other's understanding	Active listening
		Confirming whether information is conveyed by eye contact and other means
Attractive presentations	Ordered explanation	Creating a pathway and speaking in order
		Repeating the important points
	Ingenuity to attract an audience	Talking with confidence
		Using familiar words and images
		Adjusting facial expressions in response to audience
	Ingenuity to prevent boredom	Creating easy-to-understand presentation
		Appropriate speaking intervals
		Not talking persistently
		Asking a question

**Table 3:** Content categorization of Task 2 responses to “What is difficult when you tell others about an issue?”.

Category	Subcategory	Code
Difficulty identifying others	Understanding others’ comprehension level	It is difficult to confirm with eye contact
		If the others’ response is weak, I cannot see their comprehension level
	Sharing interests and emotions	Sharing interest and emotion
		Keeping the others’ interest
	Differences in generation, age, environment, and extent of knowledge compared to the opponent	The meaning of words change according to generation and environment
		Difference in knowledge quantity between myself and the opponent
Difficulty with presentations	Technical difficulty	Difficulty of currently seeing by eyes and telling by sound
		While concentrating, I cannot see others' comprehension level
		"Reading aloud" is not audible
	Difficulty of content composition	It is difficult to organize stories
		It is difficult to pick out the information I want to convey
Difficulty from lack of confidence and communication gaps	Difficulty from lack of confidence	Lack of confidence in one's thoughts and opinions
		Fear of speaking and hesitation
	Miscommunication between myself and the opponent	Possibility of misunderstanding
		Understanding varies depending on the person
		Prejudice of speaker/listener
